# Analysis of well-annotated next-generation sequencing data reveals increasing cases of SARS-CoV-2 reinfection with Omicron

**DOI:** 10.1038/s42003-023-04687-4

**Published:** 2023-03-18

**Authors:** Scott Burkholz, Michael Rubsamen, Luke Blankenberg, Richard T. Carback, Daria Mochly-Rosen, Paul E. Harris

**Affiliations:** 1 Flow Pharma, Inc., Warrensville Heights, OH USA; 2 Case Western Reserve University, Cleveland, OH USA; 3 Texas A&M University, College Station, TX USA; 4 Stanford University School of Medicine, Department of Chemical and Systems Biology, Stanford, CA USA; 5 Columbia University, Department of Medicine, College of Physicians and Surgeons, New York, NY USA

**Keywords:** Viral infection, Genetic databases

## Abstract

SARS-CoV-2 has extensively mutated creating variants of concern (VOC) resulting in global infection surges. The Omicron VOC reinfects individuals exposed to earlier variants of SARS-CoV-2 at a higher frequency than previously seen for non-Omicron VOC. An analysis of the sub-lineages associated with an Omicron primary infection and Omicron reinfection reveals that the incidence of Omicron-Omicron reinfections is occurring over a shorter time interval than seen after a primary infection with a non-Omicron VOC. Our analysis suggests that a single infection from SARS-CoV-2 may not generate the protective immunity required to defend against reinfections from emerging Omicron lineages. This analysis was made possible by Next-generation sequencing (NGS) of a Danish cohort with clinical metadata on both infections occurring in the same individual. We suggest that the continuation of COVID-19 NGS and inclusion of clinical metadata is necessary to ensure effective surveillance of SARS-CoV-2 genomics, assist in treatment and vaccine development, and guide public health recommendations.

## Abstract (web summary)

Analysis of next-generation sequencing data linked with clinical metadata from individuals reinfected with SARS-CoV-2 in a Danish cohort reveals that Omicron reinfections tend to occur at a higher frequency and within a shorter time interval compared to pre-Omicron variants.

## Introduction

The World Health Organization has designated five variants of concern (VOC) for SARS-CoV-2, the virus that causes COVID-19: Alpha, Beta, Gamma, Delta, and Omicron^1^. These VOC and emerging variants remain a significant obstacle in eliminating the SARS-CoV-2 pandemic^2^. The use of Next-generation sequencing (NGS) and accompanying metadata has allowed for a greater understanding of how VOC spread between hosts^3–5^. The Global Initiative on Sharing Avian Influenza Data (GISAID) has cataloged over 12 million SARS-CoV-2 sequences to date^3^. Data analysis suggests that each VOC is associated with a different level of infectivity and virulence^4,5^ and that novel variants, including currently circulating variants, have the potential to reinfect hosts despite global vaccination efforts^6,7^. The most recent variant, Omicron, or B.1.1.529, was designated in November 2021 as a VOC shortly after its initial identification in South Africa and Botswana^1^. The Omicron sub-lineages of BA.1, BA.2, and BA.3 have since spread around the world, becoming the initial dominant Omicron variants, followed by BA.4 and BA.5 in the weeks following^1,8^. With the exception of an Omicron-specific vaccine recently approved for use in the UK^9^ and US^10^, DNA, RNA, and whole protein vaccines designed utilizing original strain sequences have been used as part of the effort to control the pandemic. Recent studies have examined new cases of Omicron infections that have exhibited escape from vaccine-induced neutralizing antibodies, leading to increased cases of breakthrough infections^11,12^. The virus has mutated significantly into VOC since the initial outbreak in December 2019. Vaccine-induced antibodies capable of binding to the spike protein that have neutralized previous variants appear to be less effective, and antibody titers are waning over time^13,14^. Acquired antibody-mediated immunity resulting from previous exposure to a VOC has been previously considered to be sufficient to prevent an Omicron infection, however, due to the combination of waning antibodies and variant mutations, post-COVID-19 immunity does not provide complete protection against reinfection by Omicron^15^.

Our study on the genomics of viral infection and reinfection from Omicron and its sub-lineages utilizes a Denmark-based dataset comprised of SARS-CoV-2 genomes that were analyzed via NGS. Each entry was annotated with metadata for the occurrence of a reinfection. The analysis demonstrates that the reinfections via the Omicron VOC are occurring at a higher frequency when compared to previous reinfections via a non-Omicron VOC. Further analysis of sub-lineages reveals that Omicron-Omicron reinfection is occurring over a shorter time interval compared to previous non-Omicron VOC reinfections. This suggests that the Omicron variant is more capable and faster at reinfecting hosts than we have seen thus far in the pandemic. The mutation potential driving new VOC formation and thus reinfection will need to be monitored in perpetuity to inform ongoing public health policy and develop pharmaceutical modalities. The analysis presented here is an example of research that would not be possible without this robust dataset and continued genomic monitoring. NGS of emerging viral genomes will need to continue worldwide with more detailed metadata to allow better insight into the progression of SARS-CoV-2. The union of distinct datasets conveying each infected or reinfected individual’s viral genome sequence, metadata, and medical health records will be essential for this goal to be achieved.

## Results

### SARS-CoV-2 Next-Generation Sequencing Data

**Fig. 1:**
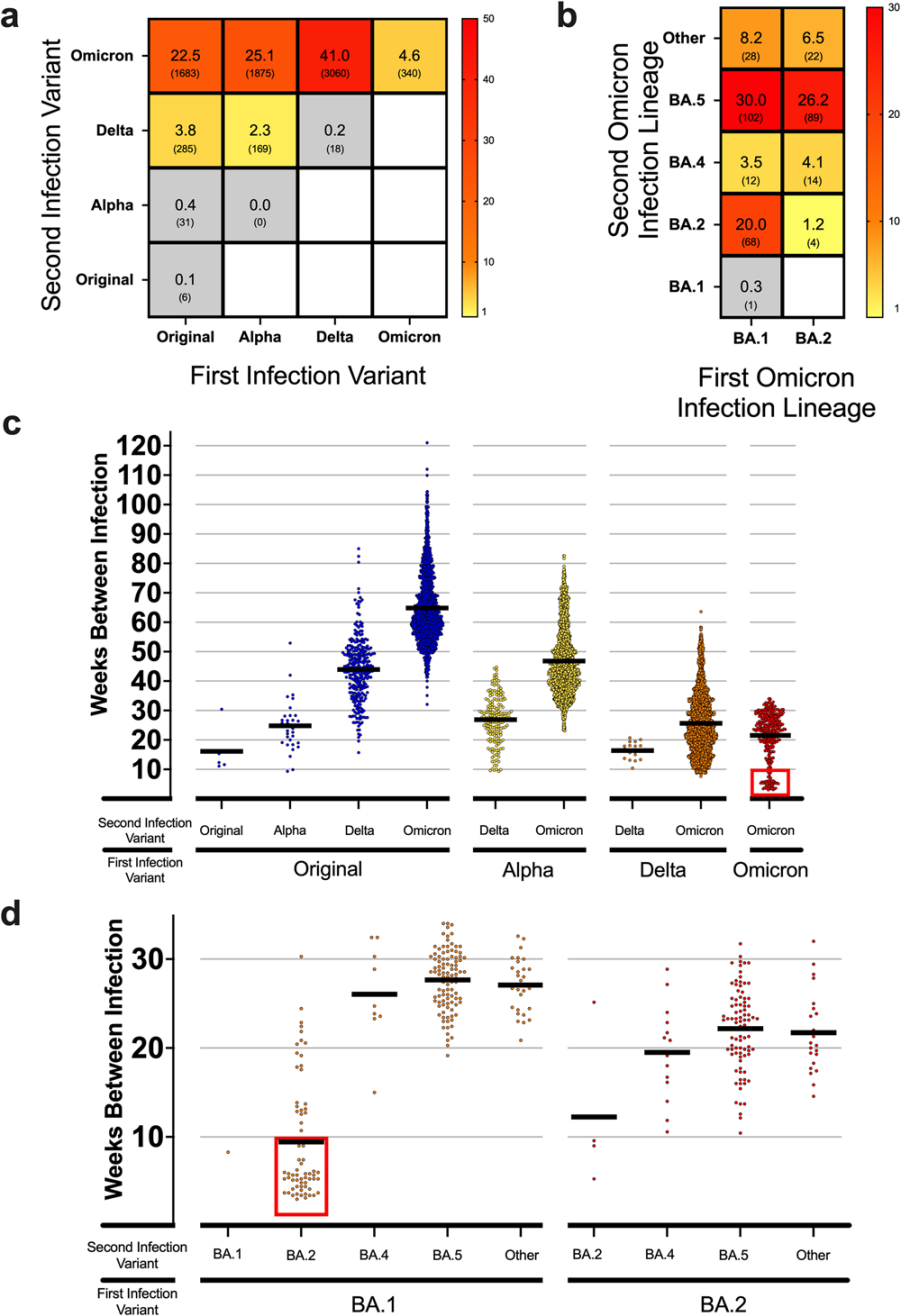
Reinfection Case Percent and Intervals by Variant. **a** Heatmap showing the frequency of total reinfections between two variants for both an initial infection and reinfection in Denmark. Raw counts shown below frequency value in parenthesis. No data was available for blank white squares. *n* = 7467 reinfection cases. **b** Heatmap showing the reinfection frequency between an initial Omicron infection and a second Omicron infection by sub-linage in Denmark. Raw counts shown below frequency value in parenthesis. No data was available for blank white squares. *n* = 340 Omicron-to-Omicron reinfection cases. **c** Scatterplot showing the time between cases (weeks) for the first and second infection of different variants in Denmark. Means of groups are shown with black bars. The red square highlights a number of early Omicron-to-Omicron cases mentioned in the text that occur before a 10-week period. *n* = 7467 reinfection cases. **d** Scatterplot for the time between cases (weeks) for Omicron-to-Omicron infections by lineage in Denmark. Means of groups are shown with black bars. The red square highlights early Omicron-to Omicron cases mentioned in the text that occur before a 10-week period for specific sub-lineage. *n* = 340 Omicron-to-Omicron reinfection cases.

**Fig. 2:**
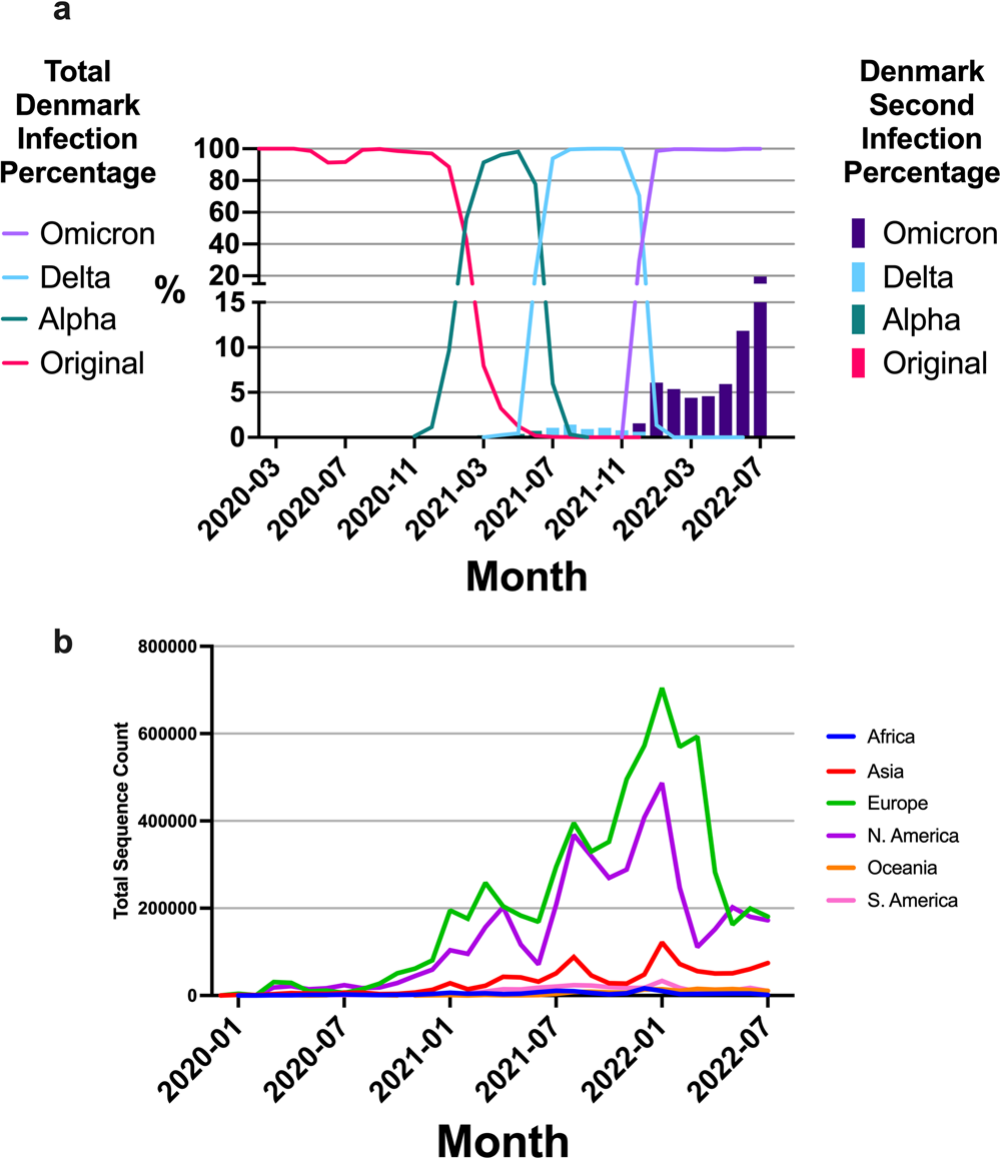
Reinfection percentages and sequencing counts. **a** Denmark sequencing percentages overtime stratified by variant represented as line plot with a bar plot overlay representing second infection percentages stratified by variant that include NGS and rtPCR data. **b** Worldwide sequencing counts stratified by continent over a period of one-month intervals. The data is also shown as percentage of cases sequenced over periods of one month in supplementary figure 2.

We report our analysis of the Danish COVID-19 Genome Consortium accessible through GISAID that examined individual cases of host reinfection across VOCs, including sub-lineages^3^. There are currently 21,708 reinfection entries available spanning March 1st 2020 to August 28th 2022. Each entry reported the exact collection date of both the initial infection and reinfection for the same individual, along with NGS sequences for the second infection’s viral genome. The primary infection and reinfection time-points were recorded as host metadata, allowing for the period between infections to be measured. A smaller portion of dataset entries (7595) reported the viral Pango lineage of both infections, in addition to the collection date, and NGS data for the initial infection and reinfection samples from the same individual. The Pango lineage nomenclature uses NGS to phylogenetically classify a virus based on its genomic composition leading to the identification of viral strains^16,17^. The majority of the dataset did not have NGS data for the initial infection, and instead relied on a reverse transcriptase polymer chain reaction (rtPCR) assay to determine if the subject was positive for SARS-CoV-2 on the reported date. The rtPCR test does not specify the viral Pango lineage, and therefore those samples were not included in the analysis shown in Fig. 1. Because the samples still contained a confirmed initial infection, they were only included in Fig. 2a, as the NGS of reinfection provided confirmation of variant Pango lineage required for analysis. A small subset of cases, 70, (<1% of total cases) had identical Pango lineages for the initial infection and reinfection. These cases were removed from the dataset because they did not satisfy the strict Pango lineage filtering methodology. This threshold was chosen because even with small nucleotide or amino acid differences within the same Pango lineage, those entries could be consistent with persistent and unresolved infection rather than reinfection^18^.

### Reinfection case percent and intervals by variant

For each NGS-characterized VOC discovered and reported with associated Pango lineage data, we found an increasing reinfection frequency favoring reinfection with the Omicron VOC (*p* < 0.0001, Chi-squared test) (Fig. 1a). 26% of individuals infected with the original viral strain showed increasingly higher reinfection frequencies with subsequent variants. Those initially infected with the Alpha variant had no cases of reinfection due to Alpha; however, increasing frequencies of reinfection from Delta (2.3%, 169 cases) and Omicron (25.1%, 1875 cases) were observed (Fig. 1a). For those initially infected with Delta, reinfection due to the Delta variant was limited (<1%, 18 cases), but 41% (3060 cases) were reported for Omicron variant reinfections. Thus far in the pandemic, reinfection within the same variant but different sub-lineages, other than for Omicron, was found to be small (0.3%, 24 cases), yet a higher number of individuals initially infected with Omicron report a reinfection due to Omicron sub-lineages (4.6%, 340 cases). There is a high frequency of reinfection with Omicron among all those reinfected since March 2020, during which time a total of 93.2% reinfections were due to Omicron. These results suggest that a primary infection with either the Original, Alpha, or Delta variant does not provide sufficient protection against reinfection, in particular for an Omicron reinfection.

In order to investigate this further, we stratified Omicron-to-Omicron reinfection cases by their major sub-lineage designation (Fig. 1b). The BA.2, BA.4, and BA.5 lineages closely share spike amino acid sequences compared to other Omicron lineages. There are only three mutational differences in the spike protein between BA.2 and both BA.4 and BA.5: del69-70, L452R, and F486V^8^. Despite this similarity, a high frequency of Omicron-to-Omicron reinfections was reported with significant difference between the observed count distribution and the expected count distribution, showing that there is a relationship between the Omicron sub-lineages for reinfection and is not due to chance (*p* < 0.0001, Chi-squared test) (Fig. 1b). Individuals initially infected with the BA.1 sub-lineage accounted for a high frequency of total Omicron-to-Omicron reinfections (62%, 211 cases) with the second infections predominately caused by BA.2 (20%, 68 cases) or BA.5 (30%, 102 cases) (Fig. 1b). Similarly, individuals initially infected with BA.2 showed comparably high frequencies of reinfection (38%, 129 cases) with BA.5 (26.2%, 89 cases) (Fig. 1b). Infections designated as “Other” in Fig. 1b did not have a common nomenclature defining the lineage. The high frequencies of reinfection suggest that the characterized difference in the BA.1 or BA.2 and BA.5 spike protein is high enough to hinder the ability of post-infection neutralizing antibodies from BA.1 or BA.2 to bind to BA.5 spike protein^12,16^. The lineages of BA.1, BA.4, and other lesser annotated Omicron variants had lower levels of re-infectivity compared to BA.2 and BA.5.

The decline of neutralizing antibodies following an infection raises a point of concern for how long natural immunity will last in a given individual. Pre-Omicron models estimated more than 90% effectiveness of initial post-infection immunity, but these estimates decrease to less than 10% after 108 weeks^19^. Figure 1c and d show the time between infections by first and second VOC seen throughout the pandemic. A stepwise trend of increasing time to reinfection is demonstrated between the different VOC due to their timing of emergence, spread, and dominance in the pandemic (Fig. 1c). In particular, Omicron-to-Omicron reinfections events appear in as little as 3 weeks after the initial infection, with a mean of 22 weeks (Fig. 1c). Of these Omicron-to-Omicron reinfections, 50 of the total 340 (14.7%) cases occur within 10 weeks of the initial infection; marked by a red box in Fig. 1c. A t-test was conducted for the significance of the time between these Omicron-to-Omicron reinfections that occur before and after 10 weeks. These two sub-groups demonstrate significance of the mean reinfection times (*p* < 0.0001, 95% CI: 17.17–20.19). This led to further analysis to determine what could be causing this significant bimodal distribution within the Omicron-Omicron reinfection group.

These reinfections from Fig. 1c are stratified by sub-lineage in Fig. 1d. The reinfection occurrence before 10 weeks, marked by a red box in Fig. 1d, is predominantly associated with BA.1 first and then reinfection with BA.2. Analysis of Variance (ANOVA) was conducted (supplementary data 1) between these groups in Fig. 1d shows significance between the difference in means for the majority of pairings. The variant sub-lineage analysis in Fig. 1 was achievable due to the availability of NGS data processed using bioinformatics to reveal the Pango lineage of samples in the dataset.

### Reinfection case percent

Using a combined dataset of both NGS and rtPCR samples as described prior, a higher number of reinfections has been reported in the past eight months (December 2021 to July 2022) compared to other VOCs in the last year (Fig. 2a). The proportion of Omicron sub-lineages against total infections in Denmark is detailed in supplementary fig. 1. During the global Omicron infection wave, Denmark had a 19.5% peak proportion of reinfection of the total sampled cases in July 2022. In comparison, a peak at 1.4% of total cases during the Delta wave were reported as reinfections. This difference highlights the high level of reinfections observed during the progression of the pandemic into the Omicron VOC. Furthermore, the data shows that when the dominant variant of infection was nearly evenly distributed at 50% between Delta and Omicron, there was not an even distribution of reinfections. The reinfections at that time, December 2021, were caused by Delta in 0.6% of cases, and by Omicron in 1.6% of cases. This is an important finding between Delta and Omicron as the likelihood of exposure was roughly the same, but the likelihood of reinfection was not.

## Discussion

Our results within this snapshot of available regional data illustrate two fundamental concepts. First, individuals are being reinfected with SARS-COV-2, and Omicron-to-Omicron reinfections appear to be occurring closer together with a higher frequency than seen for reinfections associated with previous VOC. A number of previous studies examine reinfection frequencies in SARS-CoV-2, including Omicron, and while these types of studies provide valuable insights they are often limited to PCR data only^6,7,20^, and do not designate the Pango lineage thus restricting the ability to gain insight into the pandemic. Second, there is an ongoing need for NGS with accompanying patient clinical metadata to continuously observe changes in viral infection rates and vaccine efficacy. Effective genomic tracking allows us to understand trends in viral infectivity and evaluate the current threat level of emerging VOCs.

We have seen an unprecedented level of worldwide scientific collaboration aimed at capturing viral sequences from infected individuals to build a public-facing database. This collaboration has made valuable data available to the public so that we can monitor changes in viral genomics, perform epidemiological studies, guide public health policy, and inform vaccine design^3^. Although GISAID has over 12 million sequences available at the time of this analysis, current sequencing rates are declining significantly^3^. A breakdown of NGS counts by continent is shown with recent declines in North America and Europe (Fig. 2b). This decline negatively affects the ability of our scientific community to analyze SARS-CoV-2 and COVID-19 as Pango lineage data can only be generated from NGS data. Furthermore, currently available genomic sequencing is often reported as either an initial infection or reinfection, but the initial infection and reinfection NGS data are typically not reported together for the same individual. The Denmark dataset illustrates the importance of reporting NGS data per individual, as a means to analyze reinfection risk. This is a unique cohort within the GISAID database as there were 21,708 well-annotated genomes from Denmark used in this study. Less than 2,000 additional annotated reinfection genomes were available around the World with limited levels of reinfection detail and often absent patient linkages. The size of this study, as well as the relative size of Denmark and population density, lends itself to help mitigate biases in analyses. As reinfection is becoming more common for not only a second infection, but a third and fourth infection, the data reported worldwide can provide more insight into reinfection. Additionally, chronic infections could be analyzed closer with multiple NGS analyses on samples overtime to understand how the virus evolves in a specific host.

Reinfection can be influenced by external, non-biological biases such as lockdown periods and protective measures such as masks, vaccines, and various restrictions. A national lockdown was put into place in Denmark on March 11th, 2020, with two reopening phases occurring shortly after on April 6th and on May 27th. A second national lockdown was put into place on December 16th 2020 with reopening following in two phases on February 16th and March 1st 2021. All restrictions, such as mask requirements, size limits on gatherings, and business operation restrictions were removed in September 2021. Despite reinstating these restrictions to help mitigate viral spread from December 2021 to February 2022, the 7084 Omicron reinfection cases observed over this period represented an increase from 1.5 to 6%, a reinfection level not previously seen before (Fig. 2a). Denmark became the first country in the world to halt their vaccination program, ceasing primary vaccinations as of May 15th, 2022. The reasoning stated was that 81% of the country had been vaccinated and 62% had received a third booster as of February 2022. Ending the vaccination program could have potentially caused an increase in primary infection and reinfection rates as there was still a considerable portion of unvaccinated individuals in Denmark^21–24^.

A concern in the COVID-19 pandemic is that breakthrough infections will occur in vaccinated individuals from variants and their sub-lineages due to waning neutralizing antibody titers^13,14^. We could not analyze this in our study as accompanying metadata conveying vaccine type, number of doses received, and administration date was non-existent. Vaccination metadata and more, such as age or preexisting health conditions, could allow for reinfection analysis on the level of protection or risk with more granularity on a case-by-case basis. Studies have used this type of metadata for Omicron reinfection analysis, but the NGS data defining Pango lineage is absent in lieu of rtPCR^25,26^. An analysis in Qatar was limited by rtPCR, but concluded that against BA.4 or BA.5 was present at a modest level from a previous non-Omicron infection but stronger after an Omicron infection. Individuals were still able to be reinfected regardless of the first infection variant, but interestingly this did not change much for vaccinated individuals. Vaccinated individuals were suggested to have slightly higher protection against reinfection. The findings from the study in Qatar are derived by a positive rtPCR test designed for a common Spike protein deletion correlated with the date of the dominant variant, BA.4 or BA.5. Since this deletion is present in multiple Omicron sub-lineages, BA.4, and BA.5, they cannot be separated for analysis without Pango lineage confirmation by NGS^20^.

We strongly believe that Next-generation sequencing of SARS-CoV-2 samples must continue with the addition of metadata describing the host. While the decline in sequencing is concerning, the integration of a single human’s data over multiple sources is an essential development towards discovering novel insights around COVID-19. For example, it is important to link Next-generation sequencing for viral genome identification, medical records to examine age and preexisting conditions, and metadata to track time and reinfection status.

In the case of reinfection, sequencing information characterizing both the timing between infections and viral genome composition of each infection is critical to inform accurate public health recommendations and design effective, long-lasting pharmaceuticals. Our study suggests that the reinfections with the Omicron VOC are occurring at a higher frequency and over a shorter time interval than observed for other VOC earlier during the pandemic. This has significant implications for public health policymakers. The analysis presented here is an example of research that would not be possible without this robust dataset which will need to continue NGS Worldwide and be further improved upon by the union of datasets conveying each infected or reinfected individual’s viral genome sequence, metadata, and medical health records.

Our study suggests that the reinfections with the Omicron VOC are occurring at a higher frequency and over a shorter time interval than observed for other VOC earlier during the pandemic; however, our ability to measure and quantify the viral spread has diminished exponentially due to decreased funding/ability/ to continuously sequence patient samples.

## Methods

### Data retrieval

We utilized Next-generation sequencing metadata downloaded from the Global Initiate on Sharing Avian Influenza Data (GISAID, https://gisaid.org/) on August 28th, 2022^3^. The publicly available database is updated daily and was accessed at this time for two file sets. One GISAID file contains all current metadata for over 12 million sequences in the database and is cited supplementary notes 1. A second GISAID file set contains all filtered metadata of all people in Denmark that have two linked infections reported and is cited in supplementary notes 2.

### Analysis

The metadata files are inputted into a custom-written Python script to parse the large datasets and compute values of interest. The code is available at https://gitlab.com/flowpharma/omicron-reinfection-publication and can be used to re-generate the data tables from the dataset. Entries with NGS were removed if Pango lineage for both infections is identical for the full, multi-digit designation. For these cases, it was difficult to discriminate between whether an individual experienced two distinct infections or a singular prolonged infection where they remained sick for an abnormal amount of time^27^. If the Pango lineage could not be resolved, it was also removed. After this filtering, Omicron sub-lineages are then converted to their major lineage such as BA.2.5 would become BA.2. Minor lineage raw before conversion are available in supplementary data 2. Data on the VOC Gamma and Beta as well as “Other” variants without generally accepted nomenclature are removed from the dataset or not shown where applicable due to low levels of available sequences for analysis.

### Visualization

The data tables from the resulting code were input into GraphPad Prism (GraphPad Software Inc., San Diego, CA) for visualization through various plot types and formatting options.

### Statistics and reproducibility

Calculations for statistical significance was implemented by XLSTAT (Addinsoft Inc., New York, NY) for chi-squared testing and GraphPad Prism (GraphPad Software Inc., San Diego, CA) for t-testing and ANOVA. Chi-square was performed on raw counts of cases for first and second infections as shown in Fig. 1a and b. A t-test was performed on select infection interal data, Omicron-Omicron reinfections, as mentioned in Fig. 1c. The t-test was performed under the conditions of unpaired, parametric, two-tailed, and a 95% confidence interval. The confidence interval utilizes the mean of the difference between the two groups of the t-test. ANOVA was performed for pairwise comparisons of the data in Fig. 1d. This was performed using Two-way ANOVA with a post-hoc Tukey test for multiple comparisons.

### Reporting Summary

Further information on research design is available in the Nature Portfolio Reporting Summary linked to this article.

## Supplementary information

Supplementary Information

Description of Additional Supplementary Files

Supplementary Data 1

Supplementary Data 2

Supplementary Data 3

Reporting summary

## Data Availability

The SARS-CoV-2 metadata used in the analysis described are available through Global Initiate on Sharing Avian Influenza Data (GISAID, https://gisaid.org/)^3^. Specific sequence metadata utilized are cited in supplementary notes 1 and 2. Numerical source data for the figures is available in supplementary data 3. All other data is available from the corresponding author (or other sources, as applicable) upon request.

## Code availability

The code written for the purposes described in the methods to parse the dataset is available at https://gitlab.com/flowpharma/omicron-reinfection-publication^28^.
