# Supplementary material for: Analysis of well-annotated next-generation sequencing data reveals increasing cases of SARS-CoV-2 reinfection with Omicron
Source: Commun Biol. 2023 Mar 18;6:288. doi: 10.1038/s42003-023-04687-4 (PMC10024296; doi:10.1038/s42003-023-04687-4)
Supplement: Supplementary file 1 — Supplementary Information [file 42003_2023_4687_MOESM1_ESM.pdf]

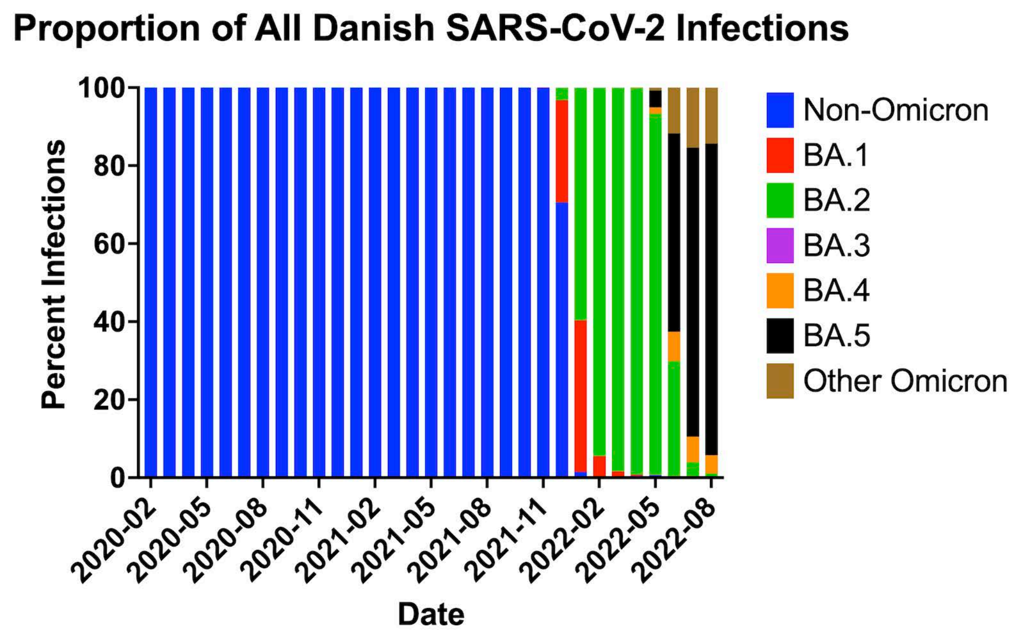

Proportion of All Danish SARS-CoV-2 Infections
Percent Infections
100
80
60
40
20
0
2020-02
2020-05
2020-08
2020-11
2021-02
2021-05
2021-08
2021-11
2022-02
2022-05
2022-08
Date
Non-Omicron
BA.1
BA.2
BA.3
BA.4
BA.5
Other Omicron

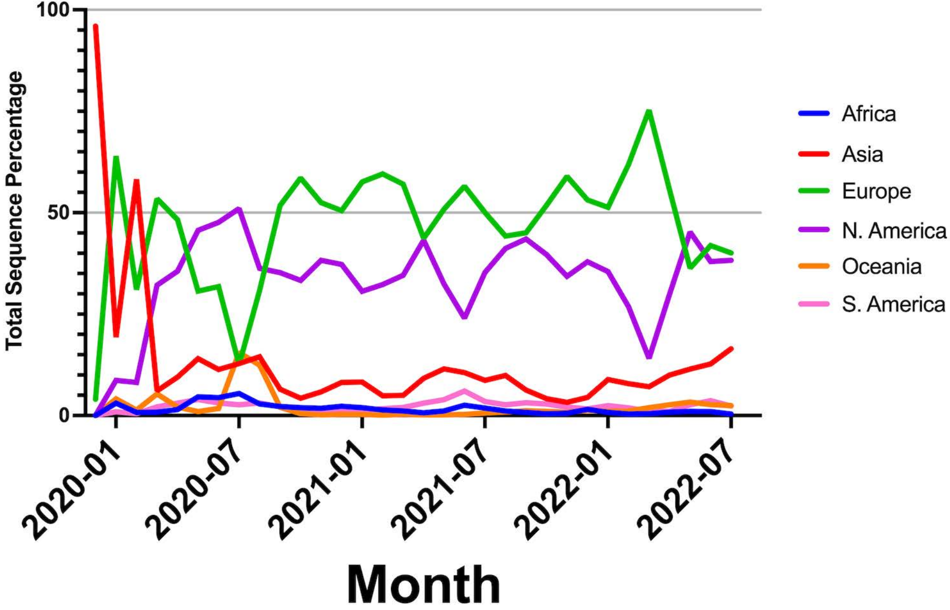

100
50
0
Total Sequence Percentage
2020-01
2020-07
2021-01
2021-07
2022-01
2022-07
Month
Africa
Asia
Europe
N. America
Oceania
S. America

Supplementary Figure 1: Proportion of Omicron sub-lineages against total infections in Denmark.

Supplementary Figure 2: Worldwide percentage of cases sequenced stratified by continent over a period of one-month intervals.

SUPPLEMENTARY NOTES 1

Data Availability

GISAID Identifier:	EPI_SET_220829mz
doi:	10.55876/gis8.220829mz

All genome sequences and associated metadata in this dataset are published in GISAID's EpiCoV database. To view the contributors of each individual sequence with details such as accession number, Virus name, Collection date, Originating Lab and Submitting Lab and the list of Authors, visit 10.55876/gis8.220829mz

Data Snapshot

- EPI_SET_220829mz is composed of 12,852,404 individual genome sequences.
- The collection dates range from 2010-12-06 to 2022-08-26;
- Data were collected in 219 countries and territories;
- All sequences in this dataset are compared relative to hCoV-19/Wuhan/WIV04/2019 (WIV04), the official reference sequence employed by GISAID (EPI_ISL_402124). Learn more at https://gisaid.org/WIV04.

SUPPLEMENTARY NOTES 2

Data Availability

GISAID Identifier: EPI_SET_220829qw
doi: 10.55876/gis8.220829qw

All genome sequences and associated metadata in this dataset are published in GISAID's EpiCoV database. To view the contributors of each individual sequence with details such as accession number, Virus name, Collection date, Originating Lab and Submitting Lab and the list of Authors, visit 10.55876/gis8.220829qw

Data Snapshot

- EPI_SET_220829qw is composed of 21,708 individual genome sequences.
- The collection dates range from 2020-08-15 to 2022-08-19;
- Data were collected in 1 countries and territories;
- All sequences in this dataset are compared relative to hCoV-19/Wuhan/WIV04/2019 (WIV04), the official reference sequence employed by GISAID (EPI_ISL_402124). Learn more at https://gisaid.org/WIV04.
