# Supplementary material for: Analysis of well-annotated next-generation sequencing data reveals increasing cases of SARS-CoV-2 reinfection with Omicron
Source: Commun Biol. 2023 Mar 18;6:288. doi: 10.1038/s42003-023-04687-4 (PMC10024296; doi:10.1038/s42003-023-04687-4)
Supplement: Supplementary file 2 — Description of Additional Supplementary Files [file 42003_2023_4687_MOESM2_ESM.pdf]

# Description of Additional Supplementary Files

**File name:** Supplementary Data 1

**Description:** – ANOVA of Omicronto-Omicron reinfection groups in figure 1d.

**File name:** Supplementary Data 2

**Description:** Denmark Omicron sublineage count table.

**File name:** Supplementary Data 3

**Description:** Data files for plots.
