# Supplementary material for: Analysis of well-annotated next-generation sequencing data reveals increasing cases of SARS-CoV-2 reinfection with Omicron
Source: Commun Biol. 2023 Mar 18;6:288. doi: 10.1038/s42003-023-04687-4 (PMC10024296; doi:10.1038/s42003-023-04687-4)
Supplement: Supplementary file 6 — Reporting summary [file 42003_2023_4687_MOESM6_ESM.pdf]

Corresponding author(s): Dr. Paul E. Harris, PhD

Last updated by author(s): Feb 20, 2023

Reporting Summary

Nature Portfolio wishes to improve the reproducibility of the work that we publish. This form provides structure for consistency and transparency in reporting. For further information on Nature Portfolio policies, see our Editorial Policies and the Editorial Policy Checklist.

Statistics

For all statistical analyses, confirm that the following items are present in the figure legend, table legend, main text, or Methods section.

n/a	Confirmed	
☐	☒	The exact sample size (*n*) for each experimental group/condition, given as a discrete number and unit of measurement
☐	☒	A statement on whether measurements were taken from distinct samples or whether the same sample was measured repeatedly
☐	☒	The statistical test(s) used AND whether they are one- or two-sided<br>*Only common tests should be described solely by name; describe more complex techniques in the Methods section.*
☒	☐	A description of all covariates tested
☐	☒	A description of any assumptions or corrections, such as tests of normality and adjustment for multiple comparisons
☐	☒	A full description of the statistical parameters including central tendency (e.g. means) or other basic estimates (e.g. regression coefficient) AND variation (e.g. standard deviation) or associated estimates of uncertainty (e.g. confidence intervals)
☐	☒	For null hypothesis testing, the test statistic (e.g. *F*, *t*, *r*) with confidence intervals, effect sizes, degrees of freedom and *P* value noted<br>*Give P values as exact values whenever suitable.*
☒	☐	For Bayesian analysis, information on the choice of priors and Markov chain Monte Carlo settings
☒	☐	For hierarchical and complex designs, identification of the appropriate level for tests and full reporting of outcomes
☒	☐	Estimates of effect sizes (e.g. Cohen's *d*, Pearson's *r*), indicating how they were calculated

Our web collection on statistics for biologists contains articles on many of the points above.

Software and code

Policy information about availability of computer code

Data collection	The SARS-CoV-2 metadata used in the analysis described are available through Global Initiate on Sharing Avian Influenza Data (GISAID, https://gisaid.org/). Specific sequence metadata utilized are cited in Supplementary Table 1 and 2. This is described in detail in the methods section for replication. Computer code to process this data is publicly available via a link in the manuscript.
Data analysis	Python 3 - Data filtering and analysis<br>GraphPad Prism 9 - Data visualization and statistics computation

For manuscripts utilizing custom algorithms or software that are central to the research but not yet described in published literature, software must be made available to editors and reviewers. We strongly encourage code deposition in a community repository (e.g. GitHub). See the Nature Portfolio guidelines for submitting code & software for further information.

Data

Policy information about availability of data

All manuscripts must include a data availability statement. This statement should provide the following information, where applicable:

- Accession codes, unique identifiers, or web links for publicly available datasets
- A description of any restrictions on data availability
- For clinical datasets or third party data, please ensure that the statement adheres to our policy

The SARS-CoV-2 metadata used in the analysis described are available through Global Initiate on Sharing Avian Influenza Data (GISAID, https://gisaid.org/). Specific

sequence metadata utilized are cited in Supplementary Table 1 and 2 with accession codes. The GISAID database access agreement prevents redistribution of this data publicly as well as addition guidelines for citations.

Human research participants

Policy information about studies involving human research participants and Sex and Gender in Research.

Reporting on sex and gender	Gender was not reported for the majority of samples used in the study, nor was it reported on as a datapoint.
Population characteristics	Population characteristics were not provided for any samples in the study, with the exception that the samples originated in Denmark.
Recruitment	Recruitment of individuals for sampling was not provided by laboratories used in the study for analysis of data.
Ethics oversight	Ethics oversight information was not provided by laboratories for this publicly available data.

Note that full information on the approval of the study protocol must also be provided in the manuscript.

Field-specific reporting

Please select the one below that is the best fit for your research. If you are not sure, read the appropriate sections before making your selection.

☒ Life sciences ☐ Behavioural & social sciences ☐ Ecological, evolutionary & environmental sciences

For a reference copy of the document with all sections, see nature.com/documents/nr-reporting-summary-flat.pdf

Life sciences study design

All studies must disclose on these points even when the disclosure is negative.

Sample size	Sample size was determined by the number of available sequences and metadata available for analysis.
Data exclusions	Data was excluded on the basis that the first infection and second infection reported an identical PANGO lineage.
Replication	Replication is not relevant to our study because the data had been collected and deposited in a database for subsequent analysis.
Randomization	Randomization is not relevant to our study because the data had been collected and deposited in a database for subsequent analysis.
Blinding	Blinding was not possible in this study as data analysis was performed on samples collected and deposited in a database.

Reporting for specific materials, systems and methods

We require information from authors about some types of materials, experimental systems and methods used in many studies. Here, indicate whether each material, system or method listed is relevant to your study. If you are not sure if a list item applies to your research, read the appropriate section before selecting a response.

Materials & experimental systems

n/a	Involved in the study	
☒	☐	Antibodies
☒	☐	Eukaryotic cell lines
☒	☐	Palaeontology and archaeology
☒	☐	Animals and other organisms
☒	☐	Clinical data
☒	☐	Dual use research of concern

Methods

n/a	Involved in the study	
☒	☐	ChIP-seq
☒	☐	Flow cytometry
☒	☐	MRI-based neuroimaging
